# Sil: A *Streptococcus iniae* Bacteriocin with Dual Role as an Antimicrobial and an Immunomodulator That Inhibits Innate Immune Response and Promotes *S. iniae* Infection

**DOI:** 10.1371/journal.pone.0096222

**Published:** 2014-04-29

**Authors:** Mo-fei Li, Bao-cun Zhang, Jun Li, Li Sun

**Affiliations:** 1 Key Laboratory of Experimental Marine Biology, Institute of Oceanology, Chinese Academy of Sciences, Qingdao, China; 2 University of Chinese Academy of Sciences, Beijing, China; 3 School of Biological Sciences, Lake Superior State University, Sault Ste Marie, Michigan, United States of America; 4 Collaborative Innovation Center of Deep Sea Biology, Zhejiang University, Hangzhou, China; Indian Institute of Science, India

## Abstract

*Streptococcus iniae* is a Gram-positive bacterium and a severe pathogen to a wide range of economically important fish species. In addition, *S. iniae* is also a zoonotic pathogen and can cause serious infections in humans. In this study, we identified from a pathogenic *S. iniae* strain a putative bacteriocin, Sil, and examined its biological activity. Sil is composed of 101 amino acid residues and shares 35.6% overall sequence identity with the lactococcin 972 of *Lactococcus lactis*. Immunoblot analysis showed that Sil was secreted by *S. iniae* into the extracellular milieu. Purified recombinant Sil (rSil) exhibited a dose-dependent inhibitory effect on the growth of *Bacillus subtilis* but had no impact on the growths of other 16 Gram-positive bacteria and 10 Gram-negative bacteria representing 23 different bacterial species. Treatment of rSil by heating at 50°C abolished the activity of rSil. rSil bound to the surface of *B. subtilis* but induced no killing of the target cells. Cellular study revealed that rSil interacted with turbot (*Scophthalmus maximus*) head kidney monocytes and inhibited the innate immune response of the cells, which led to enhanced cellular infection of *S. iniae*. Antibody blocking of the extracellular Sil produced by *S. iniae* significantly attenuated the infectivity of *S. iniae*. Consistent with these *in vitro* observations, *in vivo* study showed that administration of turbot with rSil prior to *S. iniae* infection significantly increased bacterial dissemination and colonization in fish tissues. Taken together, these results indicate that Sil is a novel virulence-associated bacteriostatic and an immunoregulator that promotes *S. iniae* infection by impairing the immune defense of host fish.

## Introduction

*Streptococcus iniae* is a Gram-positive bacterium and a major pathogen to a large number of farmed fish, notably rainbow trout, tilapia, sea bass, channel catfish, barramundi, Japanese flounder, and turbot [1], [2]. Heavy economic losses due to *S. iniae* infection have been reported in many countries including China [3], [4]. In addition, *S. iniae* is also a zoonotic pathogen and known to cause serious diseases in humans [1], [4]. A number of virulence-associated factors have been identified in *S. iniae*, including capsular polysaccharides, extracellular proteases, host factor-interacting proteins, and transcription regulators [5]–[15]. However, the infection mechanism of *S. iniae* is still obscure in many aspects.

Bacteriocins are ribosomally synthesized peptides or proteins with antibacterial activity. They are produced by a wide range of bacteria against bacteria of the same or closely related species [16]. Bacteriocins are generally grouped into two classes [17]. Class I bacteriocins (also called lantibiotics) are a class of small peptides (19–38 amino acids) characterized by the presence of unusual amino acids such as lanthionine, methyllanthionine, dehydroalanine, and 2-aminoisobutyric acid [18], [19]. Class II bacteriocins are non-lantibiotic peptides that are further subdivided into several subclasses [20].

Bacteriocins employ a variety of action mechanisms to kill their target cells, notably blocking cell wall synthesis by binding to cell wall precursors and disrupting membrane potential by producing pores in the cell membrane [21], [22]. Nisin, a lantibiotic bacteriocin, exhibits dual killing mechanisms. Through interaction with lipid II, an essential precursor for peptidoglycan biosynthesis, nisin can induce both pore formation and inhibition of cell wall biosynthesis [23]. In addition to targeting at cell membrane and cell wall, bacteriocins can also cause bacterial lysis by interfering with DNA, RNA, and protein metabolism [24].

Although bacteriocins were initially identified in *Escherichia coli*, most of the currently known bacteriocins are products of Gram-positive bacteria, in particular lactic acid bacteria (LAB) [25]. LAB-derived bacteriocins, e.g. nisin, have been used widely as preservatives in food industries. Other Gram-positive bacteria, such as those belonging to the *Bacillus* and *Staphylococcus* genera, are also producers of bacteriocins [18], [26]. In *Streptococcus*, bacteriocins have been discovered from *S. mutans*, *S. pyogenes*, *S. uberis*, and *S. salivarius* [27], [28]. However, to our knowledge, no bacteriocins from *S. iniae* have been reported.

In a recent study, we sequenced the complete genome of a pathogenic *S. iniae* isolate, SF1 [29]. We found that the SF1 genome contains a gene (named *sil*) that encodes a homologue of lactococcin 972, a nonlan bacteriocin produced by *Lactococcus lactis* IPLA 972 [30]. In the present study, we examined the biological activity of Sil. Our results indicate that Sil is a novel bacteriocin that possesses not only antimicrobial activity but also immunoregulatory property and promotes *S. iniae* infection through suppressing the immune response of host fish.

## Materials and Methods

### Ethics statement

Experiments involving live animals were conducted in accordance with the "Regulations for the Administration of Affairs Concerning Experimental Animals" promulgated by the State Science and Technology Commission of Shandong Province. The study was approved by the ethics committee of Institute of Oceanology, Chinese Academy of Sciences.

### Fish

Clinically healthy turbot (*Scophthalmus maximus*) (average 9.7 g) were purchased from a local fish farm and acclimatized in the laboratory for two weeks before experimental manipulation. Fish were fed daily with commercial dry pellets and maintained at 20°C in aerated seawater that was changed once daily. For tissue collection, fish were euthanized with tricaine methanesulfonate (Sigma, St. Louis, MO, USA) as described previously [31].

### Bacterial strains and culture conditions

The bacterial strains used in this study are listed in Table 1. Of these strains, *Lactobacillus acidophilus* 1.1854, *Streptococcus thermophilus* 1.1855, and *Streptococcus thermophilus* 1.2471 were cultured at 37°C in the MRS medium specified by China General Microbiological Culture Collection Center (CGMCC) (Beijing, China); *Lactobacillus acidophilus* 1.3221 was cultured in MRS medium at 28°C; *Streptococcus sanguinis* 1.2497 was cultured in Brain-heart infusion medium at 37°C. *Bacillus licheniformis* 1.106, *Bacillus licheniformis* 1.6510, *Bacillus oceanisediminis* 1.10115, *Escherichia coli* BL21(DE3) and DH5α, *Lactobacillus rhamnosus* 1.3724, *Lactobacillus rhamnosus* 1.2467, *Lactococcus lactis* 1.2470, *Micrococcus luteus* 1.8591, and *Streptococcus salivarius* 1.2498 were cultured in Luria-Bertani (LB) medium at 37°C.All other strains were cultured in LB medium at 28°C.

**Table 1:** Bacterial strains used in this study.

Strain	Source or reference
Gram-positive	
*Bacillus licheniformis* 1.106	CGMCC
*Bacillus licheniformis* 1.6510	CGMCC
*Bacillus oceanisediminis* 1.10115	CGMCC
*Bacillus subtilis* 1.460	CGMCC
*Lactobacillus acidophilus* 1.1854	CGMCC
*Lactobacillus acidophilus* 1.3221	CGMCC
*Lactobacillus rhamnosus* 1.2467	CGMCC
*Lactobacillus rhamnosus* 1.3724	CGMCC
*Lactococcus lactis* 1.2470	CGMCC
*Microbacterium profundi* 1.6777	CGMCC
*Micrococcus luteus* 1.8591	CGMCC
*Staphylococcus aureus* 1.363	CGMCC
*Streptococcus iniae* SF1	[32]
*Streptococcus sanguinis* 1.2497	CGMCC
*Streptococcus salivarius* 1.2498	CGMCC
*Streptococcus thermophilus* 1.1855	CGMCC^a^
*Streptococcus thermophilus* 1.2471	CGMCC
Gram-negative	
*Aeromonas hydrophila* 1.927	CGMCC
*Edwardsiella tarda* TX1	[33]
*Escherichia coli* BL21(DE3)	Tiangen, Beijing, China
*Escherichia coli* DH5α	Tiangen, Beijing, China
*Halomonas hydrothermalis* 1.6325	CGMCC
*Listonella anguillarum* C312	[34]
*Photobacterium damselae* 1.3753	CGMCC
*Pseudomonas fluorescens* TSS	[35]
*Vibrio harveyi* T4D	[36]
*Vibrio parahaemolyticus* 1.2164	CGMCC
*Yersinia ruckeri* 01	IHCAS^b^

a China General Microbiological Culture Collection Center.

b Institute of Hydrobiology, Chinese Academy of Sciences.

### Sequence analysis

The sequence of Sil was analyzed using the BLAST program at the National Center for Biotechnology Information (NCBI) and the Expert Protein Analysis System. The theoretical molecular mass and isoelectric point were predicted using the DNAStar software package (Madison, WI). Domain search was performed with the conserved domain search program of NCBI. Signal peptide search was performed with the SignalP (v3.0) program. Subcellular localization prediction was performed with the PSORTb v.3.0 server.

### Plasmid construction and purification of recombinant proteins

To construct pEtSil, which expresses C-terminally His-tagged Sil, the coding sequence of Sil was amplified by PCR with primers F1 (5’-GATATCATGGAATCAATTAGTGTAGCCG-3’, underlined sequence, EcoRV site) and R1 (5’-GATATCGTTAGAATAAAATCCAGTTGG-3’, underlined sequence, EcoRV site); the PCR products were ligated with the T–A cloning vector T-Simple (TransGen Biotech, Beijing, China), and the recombinant plasmid was digested with EcoRV to retrieve the *sil*-containing fragment, which was inserted into pET259 [37] at the EcoRV site.

### Purification of recombinant proteins and preparation of antisera

For purification of recombinant Sil (rSil), *E. coli* BL21(DE3) was transformed with pEtSil. For purification of recombinant Trx (rTrx), *E. coli* BL21(DE3) was transformed with pET32a (Novagen, San Diego, CA, USA). rTrx was used as a control protein in this study, because (i) it has been used widely in many studies as a protein tag and in general has no effect on the biological operations of eukaryotic and prokaryotic systems; (ii) it can be purified from *E. coli* under exactly the same conditions as those used for the purification of rSil, and therefore is a good control for rSil as far as the purification procedure is concerned. In addition to rSil and rTrx, the recombinant *E. tarda* proteins rEta2 [38] and rEt18 [39], which were used in protein-cell binding assay (see below sections entitled “Interaction of rSil with bacterial cells” and “Interaction of rSil with turbot HKM”), were also purified using the *E. coli* transformants BL21(DE3)/pETEta2 and BL21(DE3)/pEt18 respectively, that express these two proteins [38], [39]. For all purifications, the transformants were cultured in LB medium at 37°C to an OD_600_ of 0.5, and isopropyl-β-D-thiogalactopyranoside was added to the culture to a final concentration of 1 mM. The growth was continued at 18°C for 12 h, and recombinant proteins were purified using nickel-nitrilotriacetic acid columns (GE Healthcare, USA) as recommended by the manufacturer. The purified proteins were treated with Triton X-114 to remove endotoxin as reported previously [40]. After treatment, the proteins were dialyzed for 24 h against phosphate-buffered saline (PBS) and concentrated with PEG20000 (Solarbio, Beijing, China). The proteins were analyzed by sodium dodecyl sulfate-polyacrylamide gel electrophoresis (SDS-PAGE) and visualized after staining with Coomassie brilliant blue R-250. The concentrations of the proteins were determined using Protein Assay Kit (Sangon, Shanghai, China). Rat antisera against rSil and rTrx were prepared as reported previously [41].

### Immunoblot to determine the subcellular localization of Sil

*S. iniae* SF1 was cultured in LB medium to an OD_600_ of 0.5. The supernatant was collected by centrifugation and concentrated 20 times with PEG20000 (Solarbio, Beijing, China). The proteins were resolved by SDS–PAGE and transferred to a nitrocellulose membrane (Amersham, Cambridge, UK). Western blot was performed as described previously [42] with rSil antiserum (1/1000 dilution) or rTrx antiserum (1/1000 dilution).

### Antibacterial effect of rSil

The bacterial stains listed in Table 1 were cultured as described above to an OD_600_ of 0.8. The cells were washed with PBS and resuspended in fresh medium to 10^5^ CFU/ml. rSil was added to the cell culture to a final concentration of 20 µM, 40 µM, or 80 µM. PBS was added to the control cells. The cells were then cultured at 28°C or 37°C (as described above for different bacteria) for 0 h to 10 h, and cell density was measured using a spectrophotometer (Amersham, Cambridge, UK). To examine the bactericidal potential of rSil, *B. subtilis* 1.460 was suspended in PBS to 10^5^ CFU/ml, and rSil was added to the bacterial suspension to a final concentration of 80 µM. The cells were incubated at 28°C for 4 h or 8 h. After incubation, the cells were diluted in PBS and plated on LB agar plates. The plates were incubated at 28°C for 24 h, and the colonies emerged on the plates were counted. To examine heat sensitivity of rSil, the protein was treated at 50°C, 60°C, or 100°C for 15 min, and the antibacterial effect of the treated rSil was determined as above.

### Antibacterial effect of the culture supernatant of *S. iniae* SF1

*S. iniae* SF1 was cultured in LB medium to OD_600_ of 0.5, and the supernatant was concentrated 20 times as described above. The concentrated supernatant was passed through 0.22 µM microfilters to remove any residual bacterial cells. *B. subtilis* 1.460 was cultured as above to OD_600_ of 0.8 and collected by centrifugation. The cells were resuspended in 1 ml LB medium supplemented with or without 50 µl of (i) SF1 supernatant, (ii) SF1 supernatant plus rSil antiserum (1/200 dilution), or (iii) SF1 supernatant plus preimmune serum (1/200 dilution). The cells were incubated at 28°C for various hours and plated on LB agar plates. After incubation at 28°C for 24 h, the number of colonies was counted.

### Interaction of rSil with bacterial cells

*B. subtilis* 1.460 cultured as above was resuspended in PBS to 10^8^ CFU/ml. rSil, rTrx, rEta2, or rEt18 was added to the suspension to a final concentration of 80 µM. The cells were incubated at 28°C for 2 h and then centrifuged at 800 *g* for 5 min. The cells were collected, washed three times with PBS, and resuspended in PBS. Rat antiserum against rSil or rTrx was added to the cells (1/1000 dilution), and the cells were incubated at 30°C for 1 h. The cells were centrifuged, washed, and resuspended in PBS as above. Fluorescein isothiocyanate (FITC)-labeled goat anti-rat IgG (Bioss, Beijing, China) was added to the cells (1/1000 dilution), and the cells were incubated at 37°C for 1 h in the dark. The cells were centrifuged, washed with PBS, and resuspended in PBS. The cells were observed with a fluorescence microscope (Nikon E800, Japan).

### Effect of rSil1 on bacterial infection of turbot head kidney monocytes (HKM)

To prepare HKM, head kidney was taken from turbot (∼750 g) under aseptic conditions. The tissues were ground and passed through a sterile 75 mm metal mesh with PBS containing 10% fetal bovine serum (FBS). The cell suspension was centrifuged at 300 *g* for 5 min, and the pelleted cells were washed and resuspended in PBS containing 10% FBS. HKM were extracted from the cell suspension with Fish Monocyte Separation Kit (Hao Yang Biological Manufacture Co., Tianjin, China) as instructed by the manufacturer. The cells were distributed into 96-well tissue culture plates (∼10^5^ cells/well) containing L-15 medium (Thermo Scientific HyClone, Beijing, China). To examine the effect of rSil1 on bacterial infection, rSil or rTrx was added to HKM in the culture plates to a final concentration of 30 µM. For the control cells, the same volume of PBS was added. The plates were incubated at 22°C for 1 h. *S iniae* SF1 was cultured in LB medium to an OD_600_ of 0.8 and resuspended in L-15 to 10^7^ CFU/ml. One hundred microliters of *S. iniae* were added to each well of HKM. The plates were incubated at 22°C for 2 h or 4 h. After incubation, the cells were washed three times with PBS to remove uninfected bacteria. Penicillin and streptomycin (Sangon, Shanghai, China) (100 U) were added to the cells, and the cells were incubated at 28°C for 1 h to kill extracellular bacteria. The cells were then lysed by adding 100 µl of 1% Triton X-100 to each well; the lysate was diluted in LB medium and plated in triplicate on LB agar plates. The plates were incubated at 28°C for 48 h, and the colonies that emerged on the plates were counted. The genetic identity of the colonies was verified by PCR and sequencing analysis of randomly selected PCR products.

### Interaction of rSil with turbot HKM

HKM were resuspended in PBS to 10^7^ cells/ml. rSil, rTrx, rEta2, or rEt18 was added to the HKM suspension to a final concentration of 80 µM. The cells were incubated at 22°C for 2 h and then centrifuged at 300 *g* for 5 min. The cells were collected, washed three times with PBS, and resuspended in PBS. rSil antiserum was added to the cells (1/1000 dilution), and the cells were incubated at 30°C for 1 h. After incubation, the cells were centrifuged, washed, and resuspended in PBS as above. FITC-labeled goat anti-Rat IgG (Bioss, Beijing, China) was added to the cells (1/1000 dilution), and the cells were incubated at 37°C for 1 h in the dark. After incubation, the cells were centrifuged and washed with PBS as above. The cells were resuspended in PBS and observed under a fluorescence microscope (Nikon E800, Japan).

### Respiratory burst and acid phosphatase assays

rSil was added to HKM in a 96-well tissue culture plate (∼10^5^ cells/well) to a final concentration of 10 µM, 20 µM, or 30 µM. The same volume of PBS was added to the control cells. The plate was incubated at 22°C for 1 h. *S iniae* SF1 was cultured as above and resuspended in L-15 to 10^7^ CFU/ml. One hundred microliters of *S. iniae* was added to each well of HKM. The plate was incubated at 22°C for 3 h. After incubation, the cells were determined for respiratory burst as reported previously [43] and for acid phosphatase activity as follows: the cells were lysed by adding 100 µl of 1% Triton X-100 to each well and incubation at 4°C for 20 min. After incubation, acid phosphatase activity was determined using Acid Phosphatase Assay Kit (Beyotime, Beijing, China) according to manufacturer's instruction.

### Effect of rSil antibodies on *S. iniae* infection of turbot HKM

*S iniae* SF1 was cultured as above and resuspended in L-15 to 10^7^ CFU/ml. The bacterial suspension was mixed with antiserum against rSil (1/250, 1/500, and 1/1000 dilutions), rTrx (1/250 dilution), or preimmune serum (1/250 dilution). The control bacterial cells were mixed with PBS. After incubation at room temperature for 1 h, 100 µl of each of the mixes were added to HKM in 96-well culture plates. The plates were incubated at 28°C for 2 h. After incubation, uninfected bacteria were removed as above. The cells were lysed, and bacterial recovery was determined as above.

### Effect of rSil on *S. iniae* infection under *in vivo* conditions

Turbot were randomly divided into three groups (20 fish per group) and injected intraperitoneally with 50 µg rSil1, rTrx, or PBS. At 4 h post-injection, turbot in each group were infected via intraperitoneal injection with 100 µl *S. iniae* SF1 that had been cultured as above and resuspended in PBS to 1×10^7^ CFU/ml. At 6 h, 12 h, and 24 h post-infection, kidney and spleen were aseptically taken from the fish (five per time point). The tissues were weighed and homogenized in a glass homogenizer containing PBS. The homogenates were serially diluted and plated in triplicate on LB agar plates. The plates were incubated as above, and bacterial recovery was determined as above.

### Statistical analysis

Statistical analyses were performed using analysis of variance (ANOVA) in the SPSS 18.0 package (SPSS Inc., Chicago, IL, USA). All experiments were performed at least three times, and the results are shown as means plus or minus standard errors of the means (SEM). In all cases, significance was defined as *P*<0.05.

## Results

### Sequence of Sil

Sil is composed of 101 amino acid residues. It possesses a putative signal peptide (formed by the N-terminal 25 residues) and was predicted to be an extracellular protein. Conserved domain search indicated that Sil is a member of the lactococcin 972 family (Figure S1A). A search of the *S. iniae* SF1 genome sequence (GenBank accession no. CP005941) showed that, like lactococcin 972, which is genetically closely linked to putative ABC transporters, the *sil* gene (named lactococcin 972 in the genome) is followed immediately by the genes encoding a bacteriocin-associated integral membrane protein and an ABC transporter ATP-binding protein (Figure S1B). Blast analysis identified one close homologue to Sil, i.e., lactococcin 972 of *Lactococcus lactis*, which shares 35.6% overall sequence identity with Sil (Figure S2).

### Extracellular production of Sil

**Figure 1:**
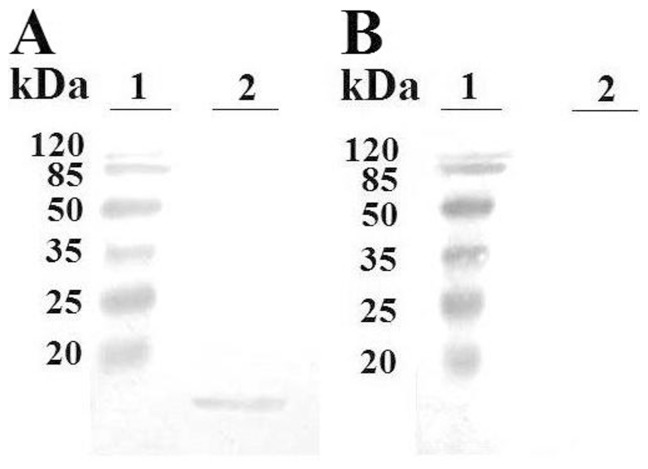
Extracellular production of Sil. *Streptococcus iniae* SF1 was cultured in LB medium to mid-logarithmic phase, and extracellular proteins were subjected to immunoblot with antibodies against rSil (A) or rTrx (B). Lane 1 of both panels, protein markers.

To facilitate functional and subcellular localization studies, rSil was purified from *E. coli* as a His-tagged protein (Figure S3) and used for antibody preparation. To examine whether, as predicted, Sil was localized in the extracellular milieu, *S. iniae* SF1 was cultured in LB medium to mid-logarithmic phase, and the proteins in the culture supernatant were subjected to Western blot with antibodies against rSil. The results showed that Sil was detected in the supernatant by anti-rSil antibodies but not by antibodies against rTrx, a His-tagged protein purified under the same condition as rSil (Fig. 1). The production level of Sil protein in the culture supernatant of SF1 was estimated by Western blot, which showed that at OD_600_ of 0.5, the amount of Sil produced by 2.4×10^7^ SF1 was approximately 1 µg (Figure S4).

### Antibacterial effect of rSil

**Figure 2:**
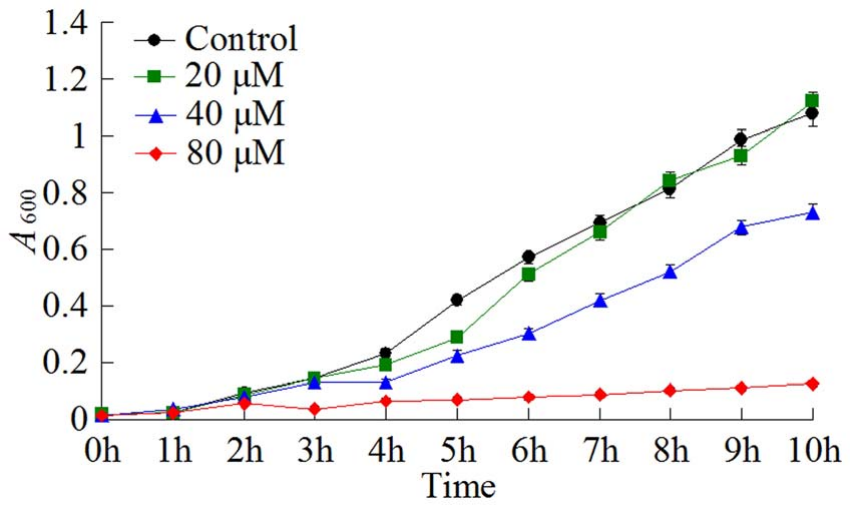
Antibacterial effect of rSil. *Bacillus subtilis* was incubated with different concentrations of rSil or PBS (control) for various hours, and cell density at each time point was determined by measuring absorbance at OD_600_. Data are the means of three independent assays and presented as means ± SEM.

To examine whether rSil possessed any antibacterial activity, rSil was incubated with 27 different bacteria (17 Gram-positive and 10 Gram-negative), which represent 23 bacterial species (13 Gram-positive species and 10 Gram-negative species). Subsequent growth analysis showed that rSil inhibited the growth of *B. subtilis* in a manner that depended on the dose of the protein (Fig. 2). In contrast, rSil had no apparent effect on the growth of all other tested bacteria (data not shown). When rSil was treated at 50°C, 60°C, or 100°C for 15 min, no antibacterial effect on *B. subtilis* was detected. To examine whether rSil was bactericidal against *B. subtilis*, the bacterium was incubated with rSil at a high dose (80 µM) for 4 h or 8 h, and the survival rate of the cells was determined by plate count. The results showed that at both examined time points, the bacterial survival rates were ∼100%, suggesting that rSil is not bactericidal.

### Antibacterial effect of the culture supernatant of *S. iniae* SF1

**Figure 3:**
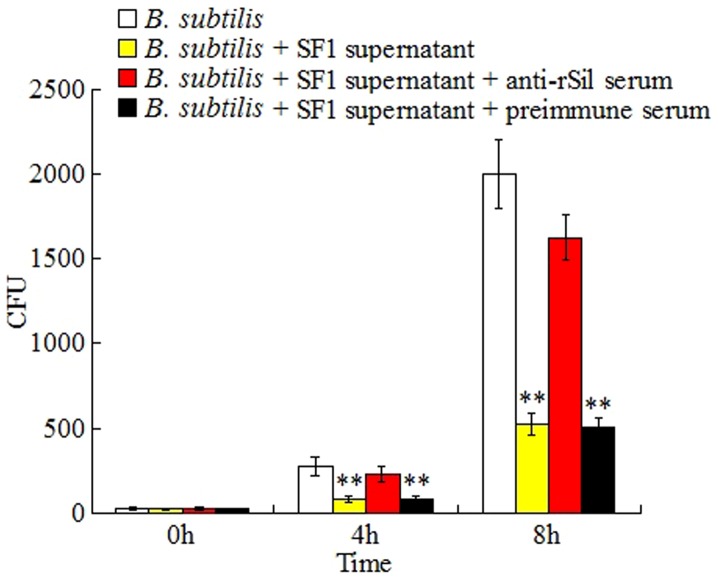
Effect of the culture supernatant of *Streptococcus iniae* SF1 on the growth of *Bacillus subtilis*. *B. subtilis* was mixed with or without the culture supernatant of *S. iniae* SF1 in the presence or absence of anti-rSil serum or preimmune serum. The mixture was incubated in LB medium for various hours, and the number of bacterial cells was determined by plate count. Data are the means of three independent assays and presented as means ± SEM.

Since, as shown above, Sil was secreted into the extracellular milieu, we examined whether the extracellular Sil had any antibacterial activity. For this purpose, the culture supernatant of *S. iniae* SF1 together with or without rSil antiserum was added to the cell culture of *B. subtilis*. The cells were then incubated under normal conditions for 4 h and 8 h. Subsequent plate count showed that although the numbers of *B. subtilis* increased with time in all cases, addition of *S. iniae* SF1 supernatant drastically decreased the number of *B. subtilis*, and that this decreasing effect of SF1 supernatant was abolished in the presence of rSil antiserum but not in the presence of preimmune serum (Fig. 3).

### Binding of rSil to target bacterial cells

**Figure 4:**
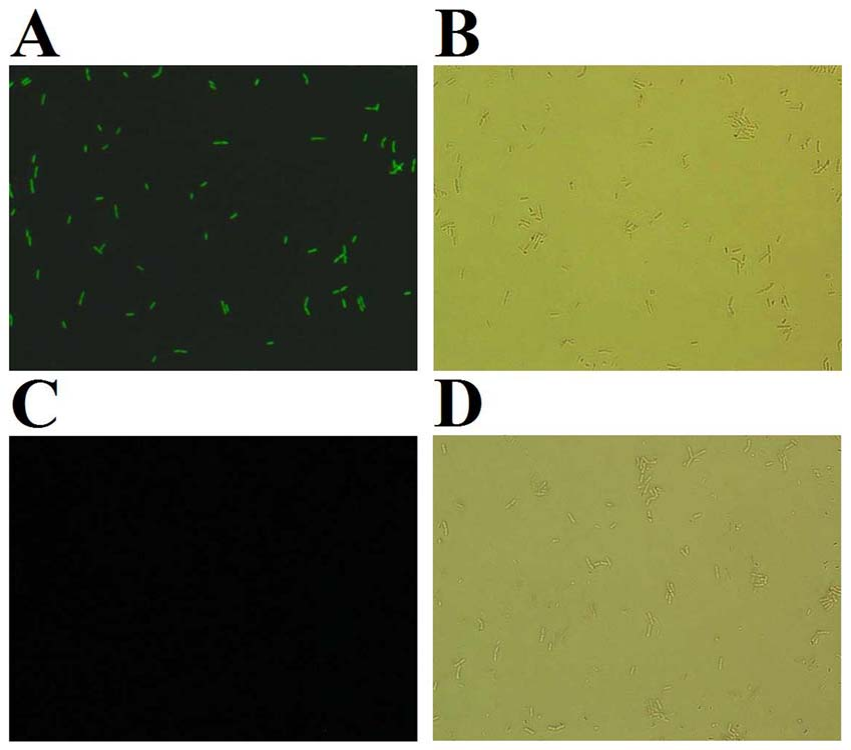
Binding of rSil to target bacterial cells. Bacillus subtilis was incubated with rSil (A and B) or rTrx (C and D), and the cell-bound protein was detected with FITC-labeled antibodies and observed under a microscope with (A and C) or without (B and D) fluorescence. Images were taken at 200 × total magnification.

To examine potential interaction between rSil and the target bacterial cells, *B. subtilis*, which is rSil-susceptible, and *Staphylococcus aureus*, which is rSil-resistant, were incubated with rSil or the control protein rTrx. The cell-bound protein was detected with FITC-labeled antibodies and observed under a fluorescence microscopy. In addition, to rule out the possibility that binding was due to nonspecific cationic interactions between the protein and the cells, the binding assay was also conducted with two more recombinant proteins, i.e. the *E. tarda* proteins rEta2 [38] and rEt18 [39] expressed in and purified from *E. coli* as rSil. Like rSil, which has an isoelectric point (pI) of 8.4, rEta2 and rEt18 also have high pI values (9.5 and 9.6 respectively) and are positively charged at physiological pH. The results showed that fluorescence was detected in *B. subtilis* treated with rSil but not in *B. subtilis* treated with rTrx, rEta2, or Et18 (Fig. 4 and data not shown). In contrast, no binding between rSil and *S. aureus* was observed (Figure S5).

### Interaction of rSil with turbot head kidney monocytes (HKM) and its effect on cellular immune defense

**Figure 5:**
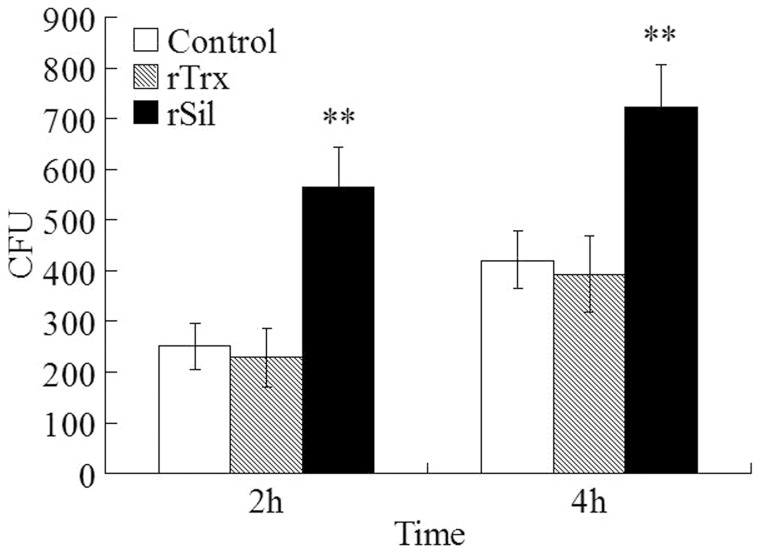
Effect of rSil on *Streptococcus iniae* infection of fish cells. Turbot head kidney monocytes were treated with or without (control) rSil or rTrx and then infected with *S. iniae* for 2 h and 4 h, and bacterial infection was determined by plate count. Data are presented as means±SEM (N = 3). ^**^ *P*<0.01.

**Figure 6:**
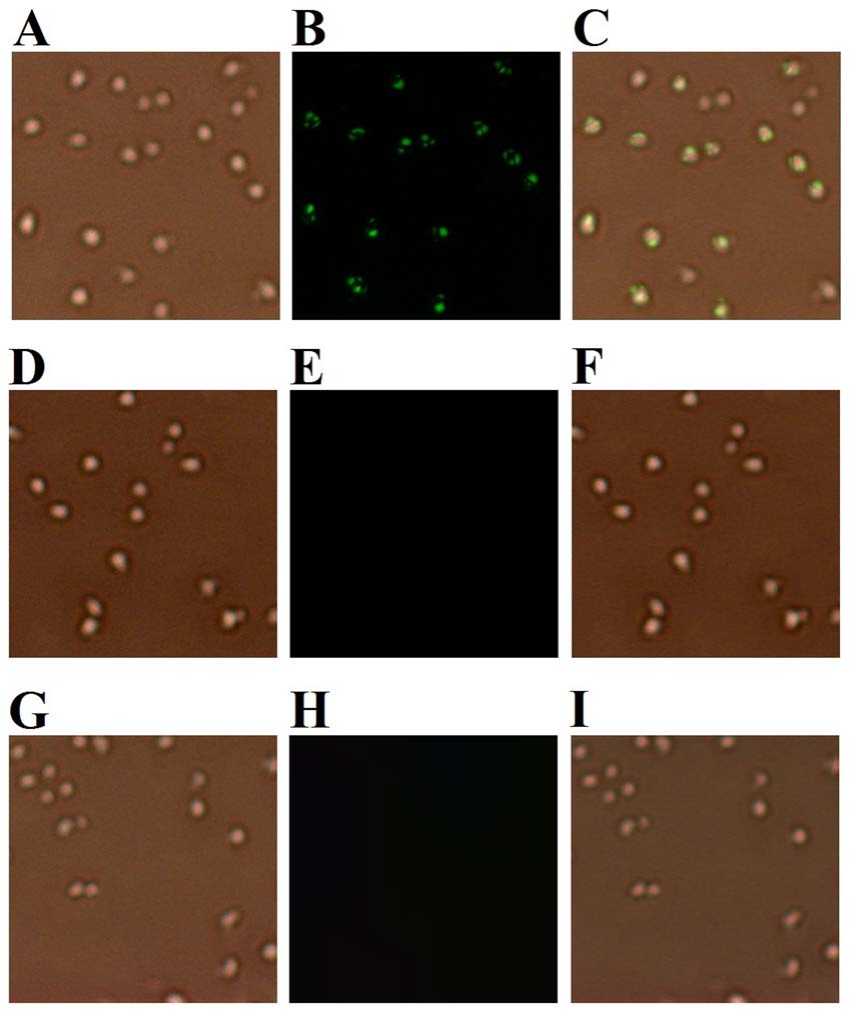
Binding of rSil to turbot head kidney monocytes (HKM). HKM were incubated with rSil (A and B), rTrx (D and E), or Eta2 (G and H), and the cell-bound protein was detected with FITC-labeled antibodies. The cells were observed under a microscope with (B, E, and H) or without (A, D, and G) fluorescence. C, merged image of A and B; F, merged image of D and E; I, merged image of G and H. Images were taken at 200 × total magnification.

**Figure 7:**
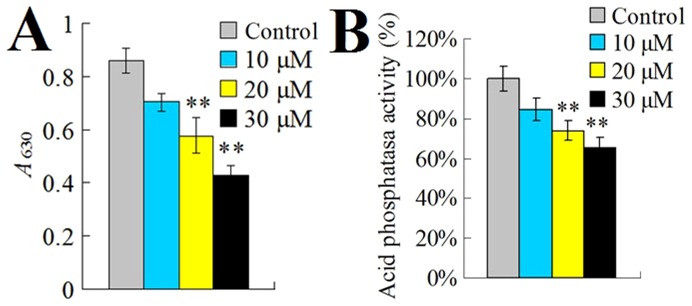
Effect of rSil on the respiratory burst and acid phosphatase activity of fish monocytes. Turbot head kidney monocytes were incubated with or without (control) different concentrations of rSil, and respiratory burst (A) and acid phosphatase activity (B) were then determined. Data are presented as means±SEM (N = 3). ^**^ *P*<0.01.

In an *in vitro* cellular infection study, we found that when *S. iniae* was incubated with turbot HKM that had been pre-treated with rSil, the number of bacterial cells succeeded in infection was significantly increased (Fig. 5). In contrast, *S. iniae* infection of HKM treated with rTrx was comparable to that of the untreated control cells. Since, as observed above, rSil had no direct effect on *S. iniae*, we wondered whether rSil could affect the activity of HKM and thereby cause enhanced bacterial infection. To investigate this question, we first examined whether there was any direct physical interaction between rSil and HKM. We found that, as revealed by immunofluorescence microscopy, rSil was indeed able to bind HKM, whereas the Sil-irrelevant proteins rEta2 and rEt18 failed to do so (Fig. 6 and data not shown). We next examined the effect of rSil-HKM interaction on the immune activity of the cells. For this purpose, HKM were treated with different concentrations of rSil and examined for respiratory burst and acid phosphatase activity. The results showed that compared to untreated cells, cells treated with rSil exhibited significantly reduced respiratory burst and acid phosphatase activity in a manner that was dependent on the dose of rSil (Fig. 7). In contrast, the levels of respiratory burst and phosphatase activity in cells treated with rTrx were comparable to those of untreated control cells (data not shown).

### Effect of rSil antibodies on *S. iniae* infection

**Figure 8:**
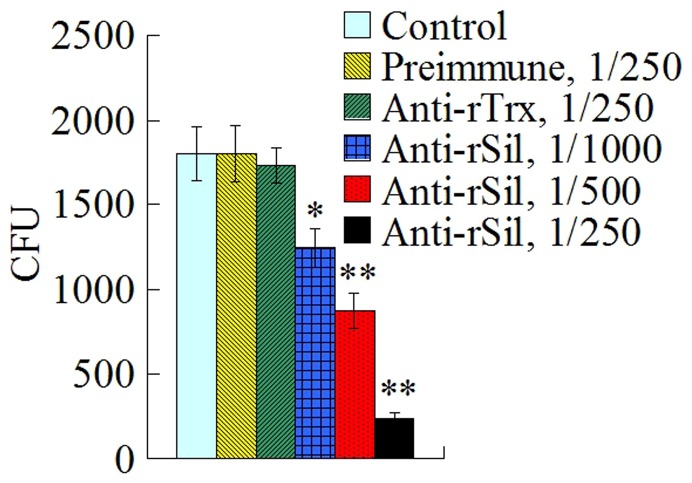
Effect of rSil antibodies on *Streptococcus iniae* infection. Turbot head kidney monocytes were incubated with *S. iniae* in the absence (control) or presence of preimmune serum or antiserum against rSil or rTrx in different dilutions, and bacterial infection was determined at 2 h after incubation. Data are presented as means±SEM (N = 3). ^**^ *P*<0.01; ^*^ *P*<0.05.

With the above results, which showed that Sil was secreted by *S. iniae* and that rSil inhibited activation of host HKM, we reasoned that antibody blocking of the extracellular Sil produced by *S. iniae* may have a negative impact on *S. iniae* infection. To examine whether or not this was true, a cellular infection study was conducted, in which HKM were incubated with *S. iniae* in the presence or absence of rSil antiserum in different dilutions. The results showed that compared to untreated cells, cells treated with rSil antiserum exhibited significantly decreased bacterial infection, in particular when the antiserum dilutions were small (Fig. 8). In contrast, the presence of preimmune serum and rTrx antiserum had no apparent effect on *S. iniae* infection.

### Effect of rSil on *S. iniae* dissemination and colonization in fish tissues

**Figure 9:**
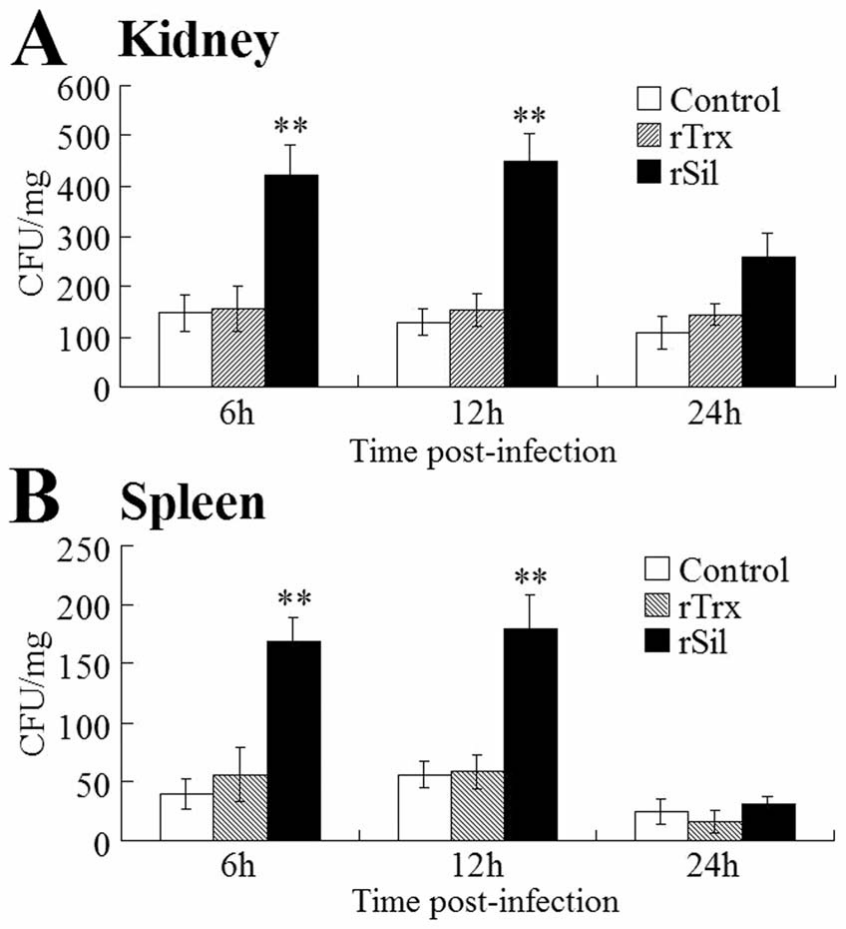
Effect of rSil on *Streptococcus iniae* infection in turbot. Turbot were administered with or without (control) rSil or rTrx before being infected with *S. iniae*. Bacterial infection into kidney (A) and spleen (B) was determined at different times post-infection. Data are presented as means±SEM (N = 3). ^**^ *P*<0.01.

So far all the biological effects of rSil were observed under *in vitro* conditions. To examine the potential *in vivo* effect of rSil, turbot were administered with rSil or rTrx before being infected with *S. iniae*. Bacterial loads in kidney and spleen were determined at 6 h, 12 h, and 24 h post-infection. The results showed that compared to untreated fish and fish treated with rTrx, fish treated with rSil exhibited significantly increased numbers of bacterial recoveries from both examined tissues at 6 h and 12 h post-infection (Fig. 9).

## Discussion

In this study, we examined the activity and biological effect of Sil, a bacteriocin from the fish pathogen *S. iniae*, under *in vitro* and *in vivo* conditions. Sil is a structural homologue of lactococcin 972 but shares low sequence identity with the latter, which suggests a molecular basis for potential functional difference between these two proteins. Consistent with the presence of a signal peptide and the predicted subcellular localization in the extracellular region, Sil was detected in the culture supernatant of *S. iniae* SF1, apparently secreted by the cultured cells. These results are in line with the structural classification of Sil being a bacteriocin.

Many bacteriocins exhibit broad-spectrum anti activities. For example, subtilomycin, a lantibiotic from *B. subtilis*, has a wide target range that includes Gram-positive and Gram-negative pathogens as well as several Candida species [44]. However, most LAB-derived bacteriocins display a target range that is limited to only closely related bacterial strains [44]–[46]. In our study, of the 27 bacteria examined, only *B. subtilis* was inhibited in growth by rSil, suggesting that rSil as a bacteriocin has a very narrow target specificity. Previous reports showed that bacteriocins with both heat-resistant and heat-sensitive properties exist, the former can maintain activity at extremely high temperatures (over 80°C) [47]–[52]. In the case of rSil, we found that it lost activity after being heated at 50°C and above, suggesting that rSil is a heat-labile protein. In addition to rSil, the culture supernatant of *S. iniae* SF1 also inhibited the growth of *B. subtilis*, which inhibition, however, was blocked by antibodies against rSil. These results indicate that the antimicrobial effect observed with rSil is most likely a reflection of the natural biological property of the Sil produced by *S. iniae*.

It is known that entry of bacteriocins into the target cell requires interaction with specific cell surface molecules, such as mannose-phosphotransferase systems and lipid II, which serve as receptors or docking sites for bacteriocins [53]. In our study, we observed specific binding of rSil to *B. subtilis*, suggesting the existence of receptor-like molecules on *B. subtilis*. It is likely that the rSil-specific receptor present in *B. subtilis* is absent in the other examined bacteria that showed resistance to rSil. Unlike most bacteriocins, which are bactericides that kill their target cells by various mechanisms, rSil inhibited the growth of *B. subtilis* but did not affect the viability of the cells. This property of rSil is apparently different from that of lactococcin 972, which blocks cell division by inhibiting septum formation, resulting in structural changes in the cell that eventually lead to cell death [54]–[56].

To date, numerous studies have been carried out on the antimicrobial activity and action mechanism of bacteriocins against the target microbial cells, however, the potential effect of bacteriocins on the immunity of animal hosts has not been investigated. In our study, we found that rSil interacted not only with target bacterial cells but also with fish HKM, and the latter caused reduction of respiratory burst and acid phosphatase activity in the immune cells. These evidences indicate that Sil is a unique bacteriocin that possesses immunoregulatory property in a negative manner. Consistent with these observations, treatment of HKM with rSil significantly weakened cellular defense against *S. iniae* infection, which is most likely due to the inability of HKM to elicit a full-scale immune response in the presence of rSil. These results point to a role of Sil as a virulence factor that promotes infection of the bacteriocin-producer strain by repressing host fish immunity. This conclusion was corroborated by the observation that antibody blocking of the extracellular Sil produced by *S. iniae* significantly impaired *S. iniae* invasion into HKM.

Although most investigations of bacteriocin activity were performed *in vitro*, some *in vivo* studies suggest application potentials of bacteriocins as antimicrobials in disease control [57]. For example, studies in murine models showed that *in vivo* administration of bacteriocins provided protection against *Salmonella* and *Listeria monocytogenes* infection [58]–[60]. In addition, bacteriocin-producing probiotic *Streptococcus salivarius* as well as other bacterial species have been found to offer beneficial modulator capabilities within local microbiome through inhibitory activity against potentially deleterious bacteria [61]–[63]. However, investigations on the *in vivo* effect of bacteriocins on the infection of the bacteriocin-producer strains are scarce. In our study, we found that pre-administration of turbot with rSil before *S. iniae* infection significantly increased bacterial dissemination into tissues of the infected fish, suggesting that, as observed in the cellular studies with HKM, rSil facilitated *S. iniae* infection under *in vivo* conditions. Given its *in vitro* effect, it is possible that the *in vivo* effect of rSil is mediated through negative modulation of the fish immune response that is essential to block bacterial invasion.

In conclusion, we demonstrate in this study that Sil is a novel bacteriocin that acts as an antimicrobial and an immunomodulator, and that in the latter capacity Sil represses the innate immunity of host fish in a manner that contributes to the infectivity of *S. iniae*. As such, Sil participates in the pathogenicity of the producer strain not only through inhibition of the growth of potentially competing bacteria but, more likely, through manipulating the immune defense of the host fish. These observations add new insights to the function and working mechanism of bacteriocins, as well as indicating for the first time that immune evasion is a likely virulence strategy of *S. iniae*.

## Supporting Information

Figure S1 **Conserved domain of Sil (A) and genetic context of the** ***sil*** **gene (B).** A. The sequence of Sil was used as a query for conserved domain search with the National Center for Biotechnology Information (NCBI) Conserved Domain Search tool. B. The genes that are located immediately downstream of the *sil* gene in the genome of *Streptococcus iniae* SF1. Note, in the genome sequence, *sil* was named lactococcin 972.
(TIF)
Figure S2
**Alignment of the amino acid sequences of Sil and lactococcin 972.**
(TIF)

Figure S3 **SDS-PAGE analysis of purified recombinant proteins.** Purified rSil (lane 2) and rTrx (lane 3) were resolved by SDS-PAGE and viewed after staining with Coomassie blue. Lane 1, protein markers.
(TIF)

Figure S4 **Estimation of the extracellular production of Sil.** *Streptococcus iniae* SF1 was cultured in LB medium to OD_600_ of 0.5, and the supernatant was concentrated 20 times. Forty microliters of the concentrated supernatant (lane 2) and rSil at 10 µg, 20 µg, and 40 µg (lanes 3 to 4 respectively) were loaded onto a SDS-PAGE gel. After electrophoresis, the gel was blotted with rSil-antibodies. Lane 1, protein markers.
(TIF)

Figure S5 **Examination of potential interaction between rSil and** ***Staphylococcus aureus*** **.** *S. aureus* was incubated with rSil (A and B) or rTrx (C and D), and the cell-bound protein was detected with FITC-labeled antibodies and observed under a microscope with (A and C) or without (B and D) fluorescence. Images were taken at 200 × total magnification.
(TIF)
