# Supplementary figures and images for: Sil: A Streptococcus iniae Bacteriocin with Dual Role as an Antimicrobial and an Immunomodulator That Inhibits Innate Immune Response and Promotes S. iniae Infection
Source: PLoS One. 2014 Apr 29;9(4):e96222. doi: 10.1371/journal.pone.0096222 (PMC4004548; doi:10.1371/journal.pone.0096222)

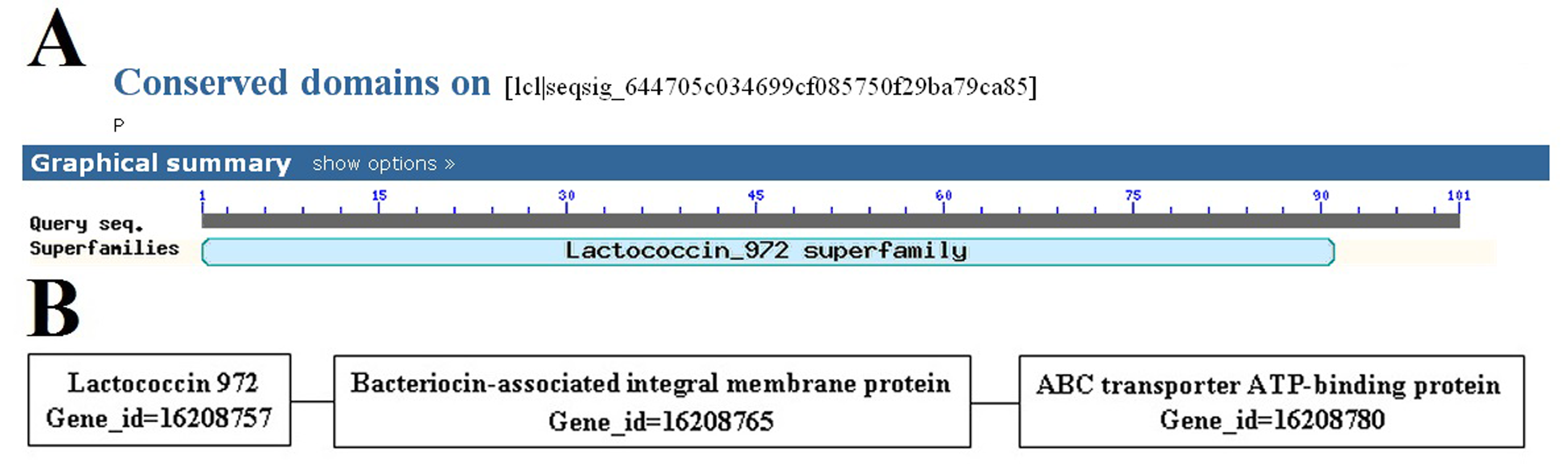

Supplement: Figure S1 — Conserved domain of Sil (A) and genetic context of the sil gene (B). A. The sequence of Sil was used as a query for conserved domain search with the National Center for Biotechnology Information (NCBI) Conserved Domain Search tool. B. The genes that are located immediately downstream of the sil gene in the genome of Streptococcus iniae SF1. Note, in the genome sequence, sil was named lactococcin 972. (TIF) Figure S2 Alignment of the amino acid sequences of Sil and lactococcin 972. (TIF) [file pone.0096222.s001.tif]

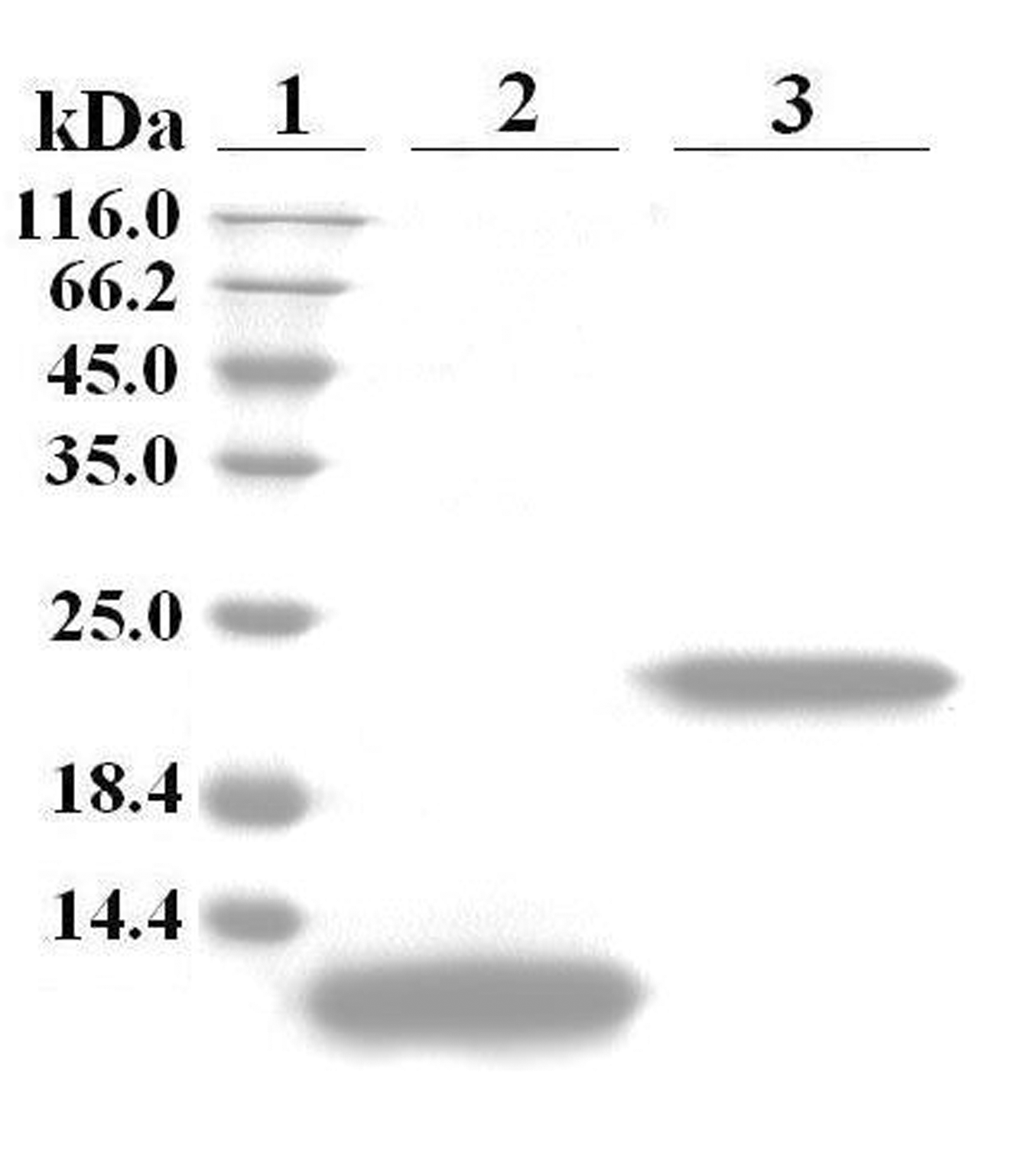

Supplement: Figure S3 — SDS-PAGE analysis of purified recombinant proteins. Purified rSil (lane 2) and rTrx (lane 3) were resolved by SDS-PAGE and viewed after staining with Coomassie blue. Lane 1, protein markers. (TIF) [file pone.0096222.s002.tif]

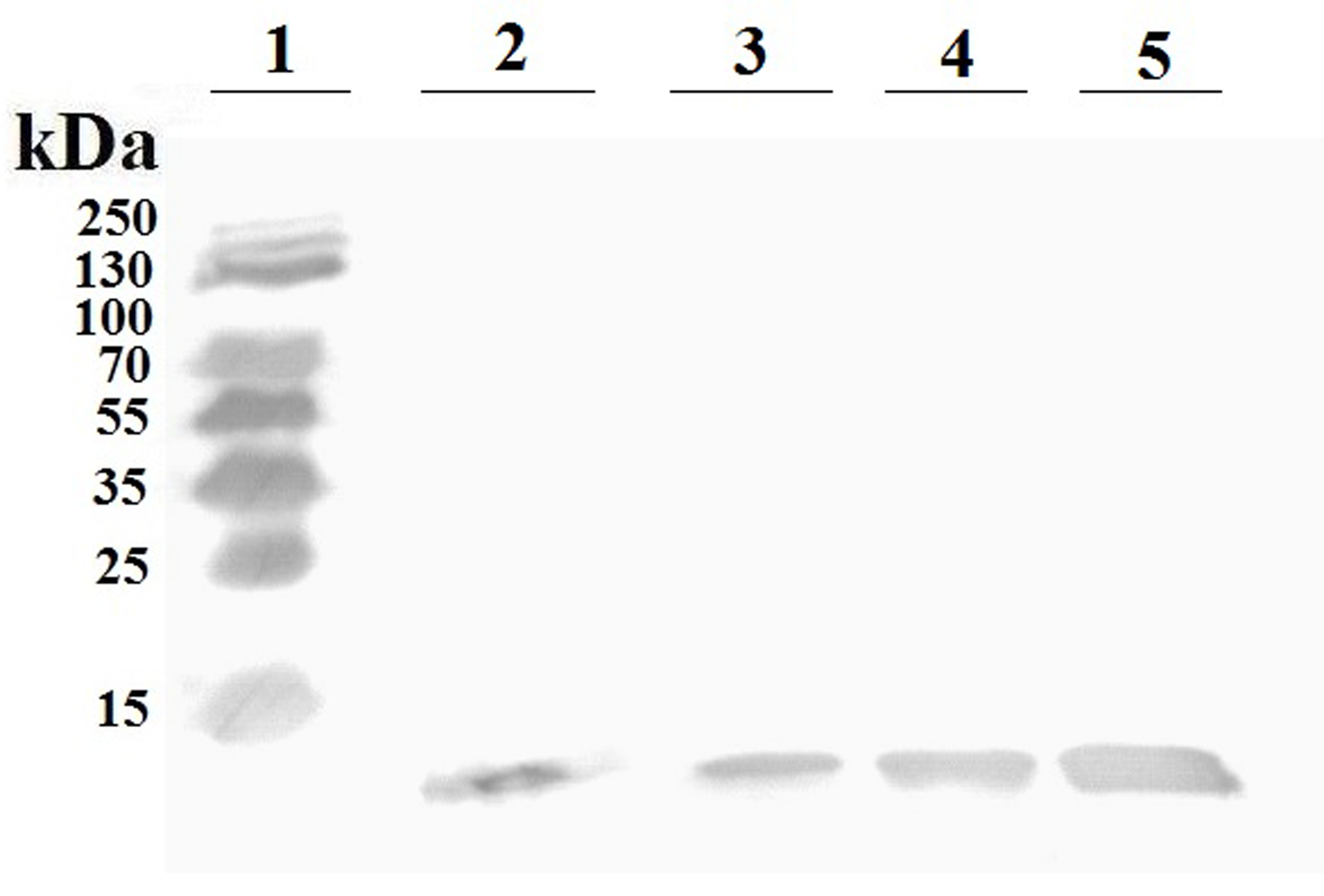

Supplement: Figure S4 — Estimation of the extracellular production of Sil. Streptococcus iniae SF1 was cultured in LB medium to OD600 of 0.5, and the supernatant was concentrated 20 times. Forty microliters of the concentrated supernatant (lane 2) and rSil at 10 µg, 20 µg, and 40 µg (lanes 3 to 4 respectively) were loaded onto a SDS-PAGE gel. After electrophoresis, the gel was blotted with rSil-antibodies. Lane 1, protein markers. (TIF) [file pone.0096222.s003.tif]

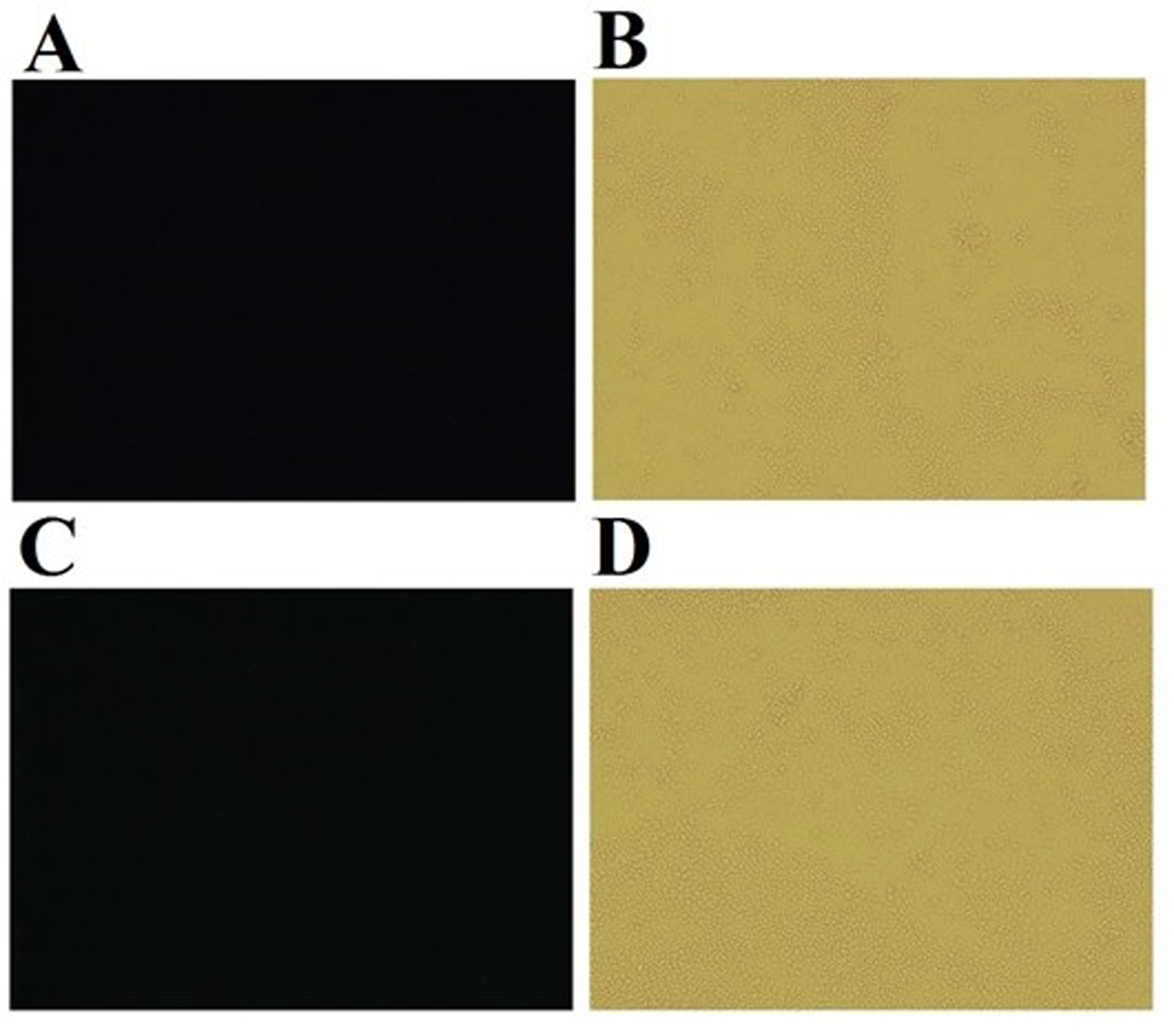

Supplement: Figure S5 — Examination of potential interaction between rSil and Staphylococcus aureus . S. aureus was incubated with rSil (A and B) or rTrx (C and D), and the cell-bound protein was detected with FITC-labeled antibodies and observed under a microscope with (A and C) or without (B and D) fluorescence. Images were taken at 200 × total magnification. (TIF) [file pone.0096222.s004.tif]
